# Long-term clinical, immunological and virological outcomes of patients on antiretroviral therapy in southern Myanmar

**DOI:** 10.1371/journal.pone.0191695

**Published:** 2018-02-08

**Authors:** Elkin Hernán Bermúdez-Aza, Sharmila Shetty, Janet Ousley, Nang Thu Thu Kyaw, Theint Thida Soe, Kyipyar Soe, Phyu Ei Mon, Kyaw Tin Tun, Iza Ciglenecki, Susanna Cristofani, Marcelo Fernandez

**Affiliations:** 1 Médecins Sans Frontières (MSF), Yangon, Myanmar; 2 Ministry of Health, Dawei, Myanmar; 3 Médecins Sans Frontières (MSF), Geneva, Switzerland; Katholieke Universiteit Leuven Rega Institute for Medical Research, BELGIUM

## Abstract

**Objective:**

To study the long-term clinical, immunological and virological outcomes among people living with HIV on antiretroviral therapy (ART) in Myanmar.

**Methods:**

A retrospective analysis of people on ART for >9 years followed by a cross-sectional survey among the patients in this group who remained on ART at the time of the survey. Routinely collected medical data established the baseline clinical and demographic characteristics for adult patients initiating ART between 2004 and 2006. Patients remaining on ART between March-August 2015 were invited to participate in a survey assessing clinical, virological, immunological, and biochemical characteristics.

**Results:**

Of 615 patients included in the retrospective analysis, 35 (6%) were lost-to-follow-up, 9 (1%) were transferred, 153 died (25%) and 418 (68%) remained active in care. Among deaths, 48 (31.4%) occurred within 3 months of ART initiation, and 81 (52.9%) within 12 months, 90.1% (n = 73) of which were initially classified as stage 3/4. Of 385 patients included in the survey, 30 (7.7%) were on second-line ART regimen; 373 (96.8%) had suppressed viral load (<250 copies/ml). The mean CD4 count was 548 cells/ mm3 (SD 234.1) after ≥9 years on treatment regardless of the CD4 group at initiation. Tuberculosis while on ART was diagnosed in 187 (48.5%); 29 (7.6%) had evidence of hepatitis B and 53 (13.9%) of hepatitis C infection.

**Conclusions:**

Appropriate immunological and virological outcomes were seen among patients on ART for ≥9 years. However, for the complete initiating cohort, high mortality was observed, especially in the first year on ART. Concerning co-infections, tuberculosis and viral hepatitis were common among this population. Our results demonstrate that good long-term outcomes are possible even for patients with advanced AIDS at ART initiation.

## Introduction

The scale up of anti-retroviral therapy (ART) in low and middle income countries (LMIC) for patients with acquired immune deficiency syndrome (AIDS) occurred in 2001–02, following the availability of generic antiretroviral drugs and roll-out of free ART programs [1]. Therefore, long-term cohort data from these settings has become available only recently. Latest data shows that long-term survival of ART patients from some LMIC have results that fall between those found in high-income countries and sub-Saharan countries [2]. In the few studies that have been published, LMIC ART cohorts are often successful in the medium-term: 84% of a South African HIV cohort, for example, remained virologically suppressed at five years [3], and 81.4% of an Indian cohort remained virologically suppressed for nearly four years [4]. Treatment outcomes and viral load (VL) results after >5 years of ART remain largely unknown in an LMIC context, as well as information about adverse metabolic complications commonly classified among the non-AIDS-defining events (NADEs) [5]. NADE causes are likely multifactorial and potentially related to demographics, lifestyle, the presence of certain co-morbidities, failing to restore CD4 to >200 cells/mm^3^, and persistent HIV-1 viral replication [6]. Research from well-resourced settings showed that among HIV-infected persons with high CD4+ cell counts, serious NADEs occur more frequently and are associated with a greater mortality risk compared to AIDS-defining events [7].

According to the latest UNAIDS data [8], access to HIV care in LMIC has seen important improvements in recent years, yet more work is still to be done. In Myanmar, the number of persons receiving ART at the end of 2015 was estimated in 106,490, representing only 47% of the need [9]. Myanmar’s HIV prevalence is estimated at only 0.6%, though rates can be ten times as high among high-risk groups such as sex workers and their clients, men who have sex with men (MSM), and injecting drug users (IDU) [10] [11]. Early reports on ART delivery in the Myanmar context were encouraging, showing that treatment at the primary care level achieved high retention in care in the short and medium-terms after ART became more accessible [12] [13], but public data on long-term outcomes remain largely unavailable. In southern Myanmar, Médecins Sans Frontières (MSF) has been providing HIV treatment and care since 2004. This study provides long-term (>9 years) clinical, immunological, and virological outcomes of a cohort of adult HIV patients and is the first of its kind in the country and one of the few produced over this length of time in the Southeast Asia region.

## Methods

### Setting and study population

With an estimated population of 1.4 million, 76% of whom live in rural areas, Tanintharyi is the southernmost region in Myanmar and covers around half of the country border with Thailand. MSF began providing HIV care and treatment in 2004 at Myitta Yeik Clinic in Dawei which is the capital of the region and was, until recently, the only HIV care provider for the region, with approximately 6000 HIV patients admitted since its inception. ART scale up by the Ministry of Health and Sports (MoHS) began in 2010, and Myanmar national HIV policy currently follows World Health Organization (WHO) recommendations, initiating treatment regardless of CD4 count.

### Retrospective analysis

All HIV-positive adults ≥18 years of age who initiated ART in Myitta Yeik clinic between September 2004 and September 2006 were included in the retrospective analysis. Data on demographic and clinical characteristics were obtained from the MSF HIV data management system (FUCHIA (Follow Up and Care of HIV infection and AIDS)). Patients were defined as lost to follow-up (LTFU) if they did not attend the clinic for more than three months after their next scheduled appointment. Baseline characteristics were analysed using standard descriptive statistics, with medians and inter-quartile ranges for continuous variables, and counts and percentages for categorical variables. Data analysis was carried out using Stata v14 (StatCorp, College Station, Texas, USA). Probability of survival was evaluated using Kaplan-Meier survival estimates, with patients alive and in care at survey enrolment, and patients’ LTFU was right-censored to the date of their last visit. Logistic regression analysed factors associated with mortality.

### Cross-sectional survey

Patients who attended to the clinic between March and August 2015 and provided consent were enrolled in the cross-sectional survey. HIV-positive children <18 years of age, pregnant women, patients who initiated ART before September 2004 or after September 2006, or patients who did not provide consent were excluded. Survey participants were asked about their medical history and the presence of current or past co-morbidities: myocardial infarction, coronary artery disease (CAD), congestive heart failure, hypertension, cerebrovascular accident, type II diabetes mellitus, cancer, renal insufficiency, tuberculosis, Hepatitis B Virus (HBV) infection, Hepatitis C Virus (HCV) infection, and cirrhosis. Cardiovascular risk factors such as tobacco smoking and alcohol consumption were also included. All eligible participants received a complete physical examination including blood pressure measurement as well as the following blood tests: complete blood count (CBC), CD4, creatinine, viral load, HCV antibody testing, HBsAg, alanine transaminase (ALAT), cholesterol and triglyceride levels, and fasting serum glucose. Threshold for viral suppression was at 250 copies/ml. For those with viral loads > 1,000 copies/ml, HIV resistance testing (genotyping) was performed. Hypertension was defined a systolic pressure ≥160 mmHg and/or a diastolic pressure ≥90mmHg for two consecutive measurements at rest. Hepatitis B infection was defined as a positive HBsAg, and hepatitis C infection as positive HCV antibody, renal insufficiency as creatinine clearance <50 ml/min, hypercholesterolemia as serum cholesterol ≥240 mg/dL, hypertriglyceridemia as serum triglyceride level >150 mg/dL, and Type II Diabetes Mellitus as one fasting serum glucose of ≥126 mg/dL, or a random plasma glucose ≥200 mg/dL.

The following investigations were conducted on site at the Myitta Yeik clinic laboratory: flow cytometry for CD4 cell counts (Clyflow II), spectrophotometry for ALAT, cholesterol and triglyceride levels, rapid testing for creatinine clearance (StatSensor), glucose (One Touch Ultra); HCV antibody testing (OraQuick® immunoassay), immunochromatography for hepatitis B surface antigen (HBsAg) (Alere Determine™). Plasma samples were tested for HIV RNA at the Mandalay Public Health Laboratory using real time PCR (RT-PCR) (GENERIC HIV Viral Load, Biocentric, Bandol, France) with a linear dynamic range of 250 (the lower limit of detection of the viral load machine) to 10 million copies/ml. Viral load testing and RT-PCR HIV resistance testing (HIV-1 reverse transcriptase genotyping) were done at Healthcare Service Solutions laboratory in Bangkok, Thailand. Genotypic drug resistance interpretation was performed using the genotypic resistance algorithms provided by Stanford HIVdb Program at https://hivdb.stanford.edu/.

The study proposal was approved by Ethics Review Committee at Department of Medical Research, MoHS, Myanmar as well as the MSF Ethical Review Board. Approval was also obtained from the local medical authorities and written informed consent was obtained from the participants for the cross-sectional survey.

## Results

### Retrospective analysis

A total of 615 patients who started ART between August 2004 and August 2006 were registered in the HIV data management system; Table 1 shows their baseline characteristics by treatment outcome. A majority were men (59.5%), with a median age of 33.2 years (IQR 29.1–39.2); 30.1% were fisherman; and 83.4% were classified as WHO stage 3 or 4 at admission. Of the 391 (63.5%) with a CD4 count available, 307 (78.5%) were below 200 cells/mm^3^. Initial ART regimen of 64.4% of patients was 3TC/D4T/NVP.

**Table 1:** Baseline characteristics of patients initiating treatment in 2004–2006 stratified by treatment outcomes, Dawei Myanmar.

	Active in Care (n = 418)	Dead (n = 153)	Lost to follow up (n = 35)	Transfer Out (n = 9)	Total (N = 615)
Gender					
Male	232 (55.5%)	104 (67.9%)	25 (71.4%)	5 (55.5%)	366 (59.5%)
Female	186 (44.4%)	49 (32%)	10 (28.6%)	4 (44.5%)	249 (40.4%)
Age					
<20y	32 (7.7%)	9 (5.9%)	1 (2.8%)	0	42 (6.8%)
20 to 29y	80 (19.1%)	30 (19.6%)	11 (31.4%)	4 (44.4%)	125 (20.3%)
30 to 39y	217 (51.9%)	69 (45.1%)	12 (34.3%)	5 (55.5%)	303 (49.3%)
40 to 49y	71 (16.9%)	33 (21.5%)	7 (20%)	0	111 (18.1%)
>49y	18 (4.3%)	12 (7.8%)	4 (11.4%)	0	34 (5.5%)
BMI					
<18.5	243 (58.1%)	47 (30.7%)	18 (51.5%)	4 (44.4%)	312 (50.7%)
18.5 to 24.9	166 (39.7%)	36 (23.5%)	8 (22.8%)	4 (44.4%)	214 (34.8%)
≥25	8 (1.9%)	2 (1.3%)	1 (2.8%)	0	11 (1.8%)
Not available	1 (0.2%)	68 (44.4%)	8 (22.8%)	1 (11.1%)	78 (12.7%)
Residence					
Dawei Town	142 (33.9%)	47 (30.7%)	10 (28.6%)	2 (22.2%)	201 (32.7%)
Dawei District	250 (59.8%)	101 (66%)	18 (51.4%)	6 (66.6%)	375 (60.9%)
Others	26 (6.2%)	5 (3.3%)	7 (20%)	1 (11.1%)	39 (6.3%)
Occupation					
Fisherman	118 (28.2%)	55 (35.9%)	11 (31.4%)	1 (11.1%)	185 (30.1%)
Migrant worker*	53 (12.7%)	23 (15.1%)	12 (34.3%)	3 (33.3%)	91 (14.8%)
Manual worker	98 (23.4%)	26 (16.9%)	3 (8.6%)	2 (22.2%)	129 (20.9%)
Farmer	15 (3.6%)	7 (4.6%)	1 (2.8%)	1 (11.1%)	24 (3.9%)
Others	29 (6.9%)	17 (11.1%)	4 (11.4%)	0	50 (8.1%)
Unemployed/Dependent	105 (25.1%)	25 (16.3%)	4 (11.4%)	2 (22.2%)	136 (22.1%)
Marital Status					
Single	106 (25.3%)	48 (31.4%)	6 (17.1%)	2 (22.2%)	162 (26.3%)
Married	178 (42.6%)	62 (40.5%)	21 (60%)	4 (44.4%)	265 (43.1%)
Separated	20 (4.7%)	12 (7.8%)	4 (11.4%)	2 (22.2%)	38 (6.2%)
Widow/er	114 (27.3%)	31 (20.2%)	4 (11.4%)	1 (11.1%)	150 (24.4%)
WHO stage at Entry					
1	13 (3.1%)	2 (1.3%)	0	0	11 (1.8%)
2	63 (15.1%)	16 (10.4%)	7 (20%)	0	86 (13.9%)
3	240 (57.4%)	80 (52.3%)	14 (40%)	6 (66.6%)	344 (55.9%)
4	102 (24.4%)	55 (39.3%)	14 (40%)	3 (33.3%)	174 (28.3%)
CD4 (mm3) at Entry					
Below 50	66 (15.7%)	25 (16.3%)	11 (31.4%)	1 (11.1%)	103 (16.7%)
50 to 99	62 (14.8%)	22 (14.3%)	4 (11.4%)	1 (11.1%)	89 (14.5%)
100 to 199	84 (20.1%)	21 (13.7%)	7 (20.0%)	2 (33.3%)	115 (18.7%)
200 to 349	50 (11.9%)	11 (7.2%)	0	2 (22.2%)	63 (10.2%)
350 to 499	4 (0.9%)	4(2.6%)	2 (5.7%)	0	10 (1.6%)
Above 500	5 (1.2%)	3 (1.9%)	2 (5.7%)	1 (11.1%)	11 (1.7%)
Not available	147 (35.3%)	67 (43.9%)	9 (25.7%)	1 (11.1%)	224 (36.5%)
Year of ART initiation					
2004 (from Aug)	29 (6.9%)	16 (10.4%)	1 (2.8%)	0	46 (7.5%)
2005	226 (54.1%)	80 (52.3%)	15 (42.8%)	4 (44.4%)	325 (52.8%)
2006 (up to Aug)	163 (38.9%)	57 (37.3%)	19 (54.3%)	5 (55.5%)	244 (39.7%)
First ART regimen					
3TC/D4T/NVP	272 (65.1%)	94 (61.4%)	24 (68.6%)	6 (66.6%)	396 (64.4%)
3TC/D4T/EFV	146 (34.9%)	59 (38.5%)	11 (31.4%)	3 (33.3%)	219 (35.6%)

*other than fisherman

**Fig 1:**
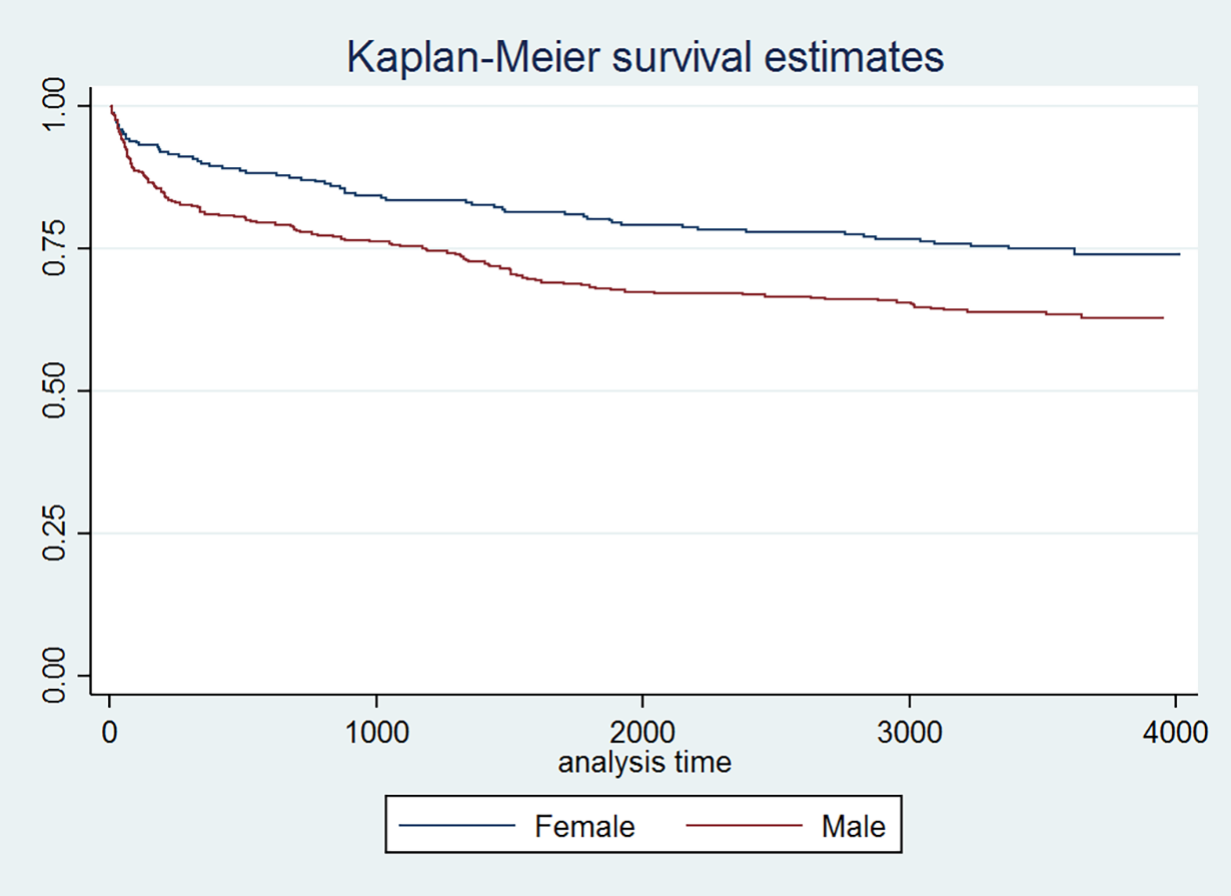
Survival curve by gender for HIV patients in an MSF follow-up program in southern Myanmar.

As of August 2015, 418 patients (68%) were still under care, 35 (6%) were lost to follow-up, 9 (1%) were transferred and 153 (25%) died. Of the 153 deaths, 48 (31.4%) died within three months of ART initiation and 81 (52.9%) died within 12 months. Of those who died within the first year of ART, 73 (90.1%) were classified as WHO stage 3 or 4 at admission. The cumulative probability of survival at 10 year among these patients was 67% (95% CI 64 to 71). The Kaplan Meier survival curve stratified by gender is shown in Fig 1.

In adjusted analysis (Table 2) mortality was associated with being >45 years of age and WHO Stage 3 and 4 at ART initiation (P<0.05).

**Table 2:** Univariate and multivariate hazard ratios (HR) for mortality.

	Active in Care N = 418 n (%)	Dead N = 153 n (%)	Univariate HR (95% CI)	HR Adjusted (95% CI)
Age at entry				
Below 30	119 (28.5)	42 (27.5)	1	1
>30 to <45	261 (62.4)	88 (57.5)	0.52 (0.32–0.83)	0.62 (0.41–0.75)
≥45	38 (9.1)	23 (15.0)	1.71 (0.91–3.24)	1.79 (1.25–2.63)
Sex				
Female	186 (44.5)	49 (32)	1	1
Male	232 (55.5)	104 (67.9)	1.65 (1.22–2.23)	1.91 (0.91–1.94)
Stage at Entry				
1 or 2	72 (17.1)	18 (11.8)	1	1
3 or 4	346 (82.8)	135 (88.2)	1.91 (0.81–2.72)	1.68 (1.33–2.59)
BMI				
<18.5	243 (48.6)	47 (55.3)	1.22 (0.23–6.27)	1.19 (0.36–2.35)
18.5 to 24.9	166 (39.5)	36(42.4)	1.13 (0.21–5.61)	1.09 (0.44–1.97)
≥25	8 (1.9)	2 (1.3)	1	1

### Cross-sectional analysis

Of the 418 patients under care in August 2015, 33 (7.9%) patients were excluded because not available (n = 23) or declined to participate (n = 10), then 385 (92.1%) patients were included in the cross-sectional survey (Table 3). Most patients were male (57.4%), of Bamar ethnicity (92.4%), and manual labourers by profession (51.2%). Most (74%) were >40 years of age (mean 44.4, SD 7.9). More than 80% of patients had a CD4 count >350/mm^3^, and 67.6% had a CD4 count >500/mm^3^. ART regimen was changed for 47% of patients to a TDF (Tenofovir disoproxil fumarate) based regimen over the study period (44% were still on that regime at time of analysis). Thirty (7.8%) patients changed to second line treatment after an average of 7.6 years on first line. None of the patients were on third line treatment. ART-related toxicity was the primary reason for any drug regime change in 212 patients (55.1%).

**Table 3:** Characteristics of patients who were included in the survey distributed by gender.

	Female (n = 164)	Male (n = 221)	Total (n = 385)
Age			
<20y	0	1 (0.5%)	1 (0.3%)
20 to 29y	5 (3%)	2 (0.9%)	7 (1.8%)
20 to 39y	37 (22.6%)	55 (24.8%)	92 (23.8%)
40 to 49y	76 (46.3%)	125 (56.6%)	201 (52.2%)
>49y	46 (28%)	38 (17.2%)	84 (21.8%)
Ethnicity			
Bamar	150 (91.4%)	206 (93.2%)	356 (92.4%)
Others	14 (8.5%)	15 (6.7%)	29 (7.5%)
Occupation			
Manual Worker	86 (52.5%)	111 (50.2%)	197 (51.2%)
Farmer/Fisherman	8 (4.8%)	43 (19.4%)	51 (13.2%)
Others	57 (34.7%)	17 (7.7%)	74 (19.2%)
Unemployed	13 (7.9%)	50 (22.6%)	63 (16.4%)
BMI (current)			
<18.5	37 (22.6%)	49 (22.2%)	86 (22.3%
18.5 to 24.9	100 (60.9%)	156 (70.5%)	256 (66.5%)
≥25	27 (16.4%)	16 (7.2%)	43 (11.1%)
Last CD4 count (mm3)			
Below 50	0	0	0
50 to 99	1 (0.6%)	2 (0.9%)	3 (0.7%)
100 to 199	1 (0.6%)	11 (4.9%)	12 (3.1%)
200 to 349	10 (6.1%)	51 (23.1%)	61 (15.8%)
350 to 500	37 (22.6%)	63 (28.5%)	100 (25.9%)
Above 500	115 (70.1%)	94 (42.5%)	209 (54.3%)
Viral Load			
Undetectable	161 (98.1%)	212 (95.9%)	373 (96.8%)
Detectable	3 (1.8%)	9 (4.1%)	12 (3.1%)
Year of ART initiation			
2004 (from Aug)	13 (7.9%)	14 (6.3%)	27 (7%)
2005	87 (53%)	122 (55.2%)	209 (54.3%)
2006 (up to Aug)	64 (39%)	85 (38.5%)	149 (37.7%)
First ART regimen			
3TC/TDF/EFV	124 (75.6%)	124 (56.2%)	248 (64.4%)
3TC/AZT/NVP	40 (24.4%)	97 (43.8%)	137 (35.6%)
Current ART regimen			
3TC/TDF/EFV	69 (42.1%)	102 (46.2%)	171 (44.4%)
3TC/AZT/NVP	72 (43.9%)	79 (35.7%)	151 (39.2%)
Other First line	17 (10.3%)	16 (7.2%)	33 (8.6%)
Second line	6 (3.6%)	24 (10.8%)	30 (7.8%)

### Immunological characteristics

**Fig 2:**
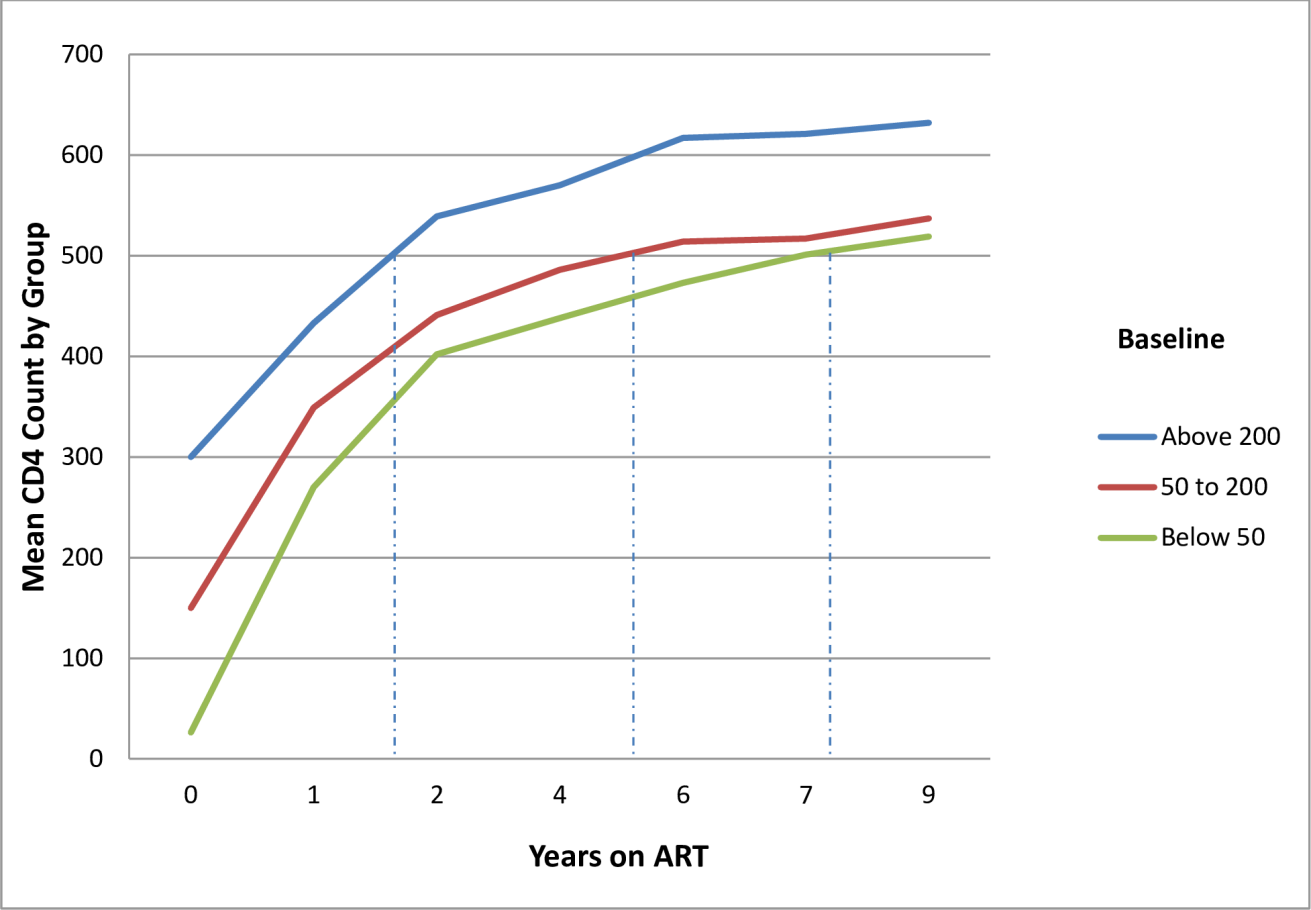
CD4 count trend over time for patients still on ART after 9 years or more, Dawei, 2015.

Fig 2 shows the mean CD4 evolution over time after ART initiation by group: initial CD4 <50/mm^3^, 50–200, and >200. The mean CD4 count was 548 cells/ mm^3^ (SD 234.1) after ≥9 years on treatment regardless of the CD4 group at initiation.

### Viral load and resistance

Of 385 patients, only 12 had a detectable VL (3.1%). Of those, 4 had VL >1000 copies/ml, all of which were then eligible for genotyping and all of whom were shown to have subtype CRF 01_AE. Three of the four isolates showed mutations for the PR region with Major: M46I, L76V, I84V; and Minor: L70F (in one patient). Two of these patients were on second line regimen. The fourth patient did not show any relevant mutations for all the regions analysed. Nucleoside/Nucleotide Reverse Transcriptase Inhibitors (NRTI) mutations included: K65R, D67N, F77L and F116Y with one sample showing no resistance-associated mutations. The most common Non-Nucleoside Reverse Transcriptase Inhibitors (NNRTIs) mutations were V90I, V179T, Y181C and G190A.

### Non-AIDS defining events, risk factors, and co-morbidities

The most common past or current co-morbidity was tuberculosis (48.5%), additionally, one patient was diagnosed with active tuberculosis at the time of assessment. Two patients were previously diagnosed with CAD, and none with myocardial infarction or cancer. Out of 373 patients with available results (Table 4), elevated levels of ALAT were seen in 141 (37.8%), with 7 of them on second line treatment; and high triglyceride levels were observed in 143 (42.8%), with 23 on second line treatment. The presence of HCV antibodies was detected in 13.7% of the participants, and HbsAg in 7.5%. Past tuberculosis infection and high triglyceride levels were more common in male patients. Among women, tobacco smoking was rare (<5%) as was alcohol consumption (2.5%). Among men these proportions were 50.2% and 85.1%, respectively.

**Table 4:** Co-morbidities and risk factors among patients participated in the survey according to gender, Dawei, 2015.

	Female (n = 164)	Male (n = 221)	Total (n = 385)
Hypertension^£^			
Yes	25 (15.2%)	44 (19.9%)	69 (17.9%)
No	139 (84.7%)	177 (80.1%)	316 (82.1%)
Tuberculosis (past infection)			
Yes	57 (34.7%)	130 (58.8%)	187 (48.5%)
No	107 (65.3%)	91 (41.2%)	198 (51.5%)
HbsAg			
Positive	10 (6.1%)	19 (8.6%)	29 (7.5%)
Negative	152 (92.7%)	197 (89.1%)	349 (90.6%)
Not available	2 (1.2%)	5 (2.3%)	7 (1.8%)
HCV antibodies			
Positive	10 (6.1%)	43 (19.5%)	53 (13.7%)
Negative	152 (92.7%)	175 (79.2%)	327 (84.9%)
Not available	2 (1.2%)	3 (1.3%)	5 (1.3%)
Cirrhosis diagnosis (Yes)	1 (0.6%)	3 (1.3%)	4 (1.1%)
DM diagnosis (Yes)	7 (4.2%)	8 (3.6%)	15 (3.8%)
Renal Insufficiency (Yes)	14 (8.5%)	17 (7.7%)	31 (8.1%)
Previous Cancer Diagnosis (Yes)	0	0	0
ALAT			
Above limit*	71 (43.3%)	70 (31.7%)	141 (36.6%)
Normal	87 (53%)	145 (65.6%)	232 (60.2%)
Not available	6 (3.6%)	6 (2.7%)	12 (3.1%)
Glucose levels			
High**	7 (4.3%)	14 (6.3%)	21 (5.5%)
Normal	153 (93.3%)	200 (90.5%)	353 (91.7%)
Not available	4 (2.4%)	7 (3.2%)	11 (2.8%)
Triglyceride levels			
High***	49 (29.8%)	94 (42.5%)	143 (37.1%)
Normal	99 (60.4%)	92 (41.6%)	191 (49.6%)
Not available	16 (9.7%)	35 (15.8%)	51 (13.2%)
Total Cholesterol levels			
High****	17 (10.3%)	5 (2.2%)	22 (5.7%)
Normal	131 (79.9%)	181 (81.9%)	312 (81%)
Not available	16 (9.7%)	35 (15.8%)	51 (13.2%)
LDL			
≤ 160 mg/dL	130 (79.3%)	182 (82.3%)	312 (81%)
>160 mg/dL	18 (10.9%)	4 (1.8%)	22 (5.7%)
Not available	16 (9.7%)	35 (15.8%)	51 (13.2%)
HDL			
≤35 mg/dL	61 (37.1%)	79 (35.7%)	140 (36.3%)
>35 mg/dL	87 (53.1%)	107 (48.4%)	194 (50.4%)
Not available	16 (9.7%)	35 (15.8%)	51 (13.2%)
Tobacco Smoking			
Never	159 (96.9%)	110 (49.7%)	269 (69.9%)
Less than daily	1 (0.6%)	10 (4.5%)	11 (2.8%)
At least once daily	4 (2.4%)	101 (45.7%)	105 (27.2%)
Alcohol consumption			
Never	160 (97.5%)	33 (14.9%)	193 (50.1%)
Less than daily	3 (1.8%)	182 (82.4%)	185 (48.1%)
At least one dose daily	1 (0.6%)	6 (2.7%)	7 (1.8%)

^£^Defined as Systolic Blood Pressure ≥140 mm Hg and/or Diastolic Blood Pressure ≥90 mm Hg

*Normal level: for males: <45U/L: for females: <34 U/L

**Defined as fasting plasma glucose ≥ 126 or random measure ≥ 200

***serum triglyceride level >150 mg/dL

****serum cholesterol ≥240 mg/dL.

## Discussion

This study is one of the first analyses of long term clinical, immunological and virological outcomes in an LMIC cohort on ART >9 years, and is one of the first to examine primary comorbidities, NADEs, and baseline HIV-related characteristics for patients initiating ART during the same period. It is the first study of its kind in Myanmar.

### Retention and mortality outcomes

Attrition in the study cohort was primarily driven by high early mortality (total: 25%, of those 52.9% within the first 12 months on ART) and correlated with advanced WHO stage at admission and older age. The exact causes of death were not known as non-active investigation was originally conceived as part of the program. The retention in care (67.9% of patients in care after ≥9 years on ART) were similar or better to cohorts studied in other LMIC settings. In Uganda (n = 559) and Haiti (n = 910), 65% and 53% of patients respectively were still on ART after 10 years, and mortality rates from these two studies were also similar: 27% of Haitian patients died while on treatment, 42% within the first 6 months and 84.2% originally presented as WHO Stage 3 or 4 [14] [15]. The Ugandan study’s cumulative death rate of 22.7% similarly had 63% of deaths occurring within one year of initiating ART. Another Myanmar study of a five-year program in Yangon, the country’s most populous city and former capital, found that 39.8% of deaths occurred six months after ART initiation [16], and multivariate analysis suggested that only ART exposure status and baseline CD4 count were independently predictive of mortality.

Cumulative LTFU in the study cohort was relatively low (5.7%) when compared with other LMIC settings. A multicentric study from across Africa and Asia [17] reported a drop in mortality (from 17% to 12%) over eight years while at the same time noting a substantial increase in LTFU (from 6% to 15% at 12 months; and 11% to 21% at 36 months).

### Immunological outcomes

Predictably, immune response improved over time for this cohort, and patients in care for >9 years achieved a mean CD4 count result >500 cells/mm^3^ regardless of baseline CD4 count, similar to positive CD4 evolution in 4–5 year study cohorts from the United States and from Africa, Latin America and other parts of Asia [18] [19] [20]. Our results, after following ART patients for nearly twice as long as most other long term studies, reaffirm that sustained immunological response to ART can be maintained in both economically developed as well as LMIC settings in Asia.

### Virological characteristics

The high proportion of ART patients who were virally supressed in our cohort speaks to the strength of the overall ART clinic and program and even compares with virological suppression rates in high income countries (HIC) and some LMIC. Successful virological outcomes in LMIC are known to be possible (>80% VL suppression in a meta-analysis of 5 year HIV programs, 70% VL suppression in a 6–7 year Nigerian cohort) and stable over time; usually with initially significantly higher rates of VL suppression in Asia than in sub-Saharan Africa, a difference that tends to disappear over time in published research [21] [22]. Descriptions of high income European cohorts show an increase in virological failure in the first 3–4 years that appears to plateau at around 1.0–1.5 per 100 person-years until year 9, with the estimated cumulative proportion of patients who remain virologically supressed at 91.4% [23]. Among a Swiss HIV cohort of 9-year ART patients (n = 3,680), 72% were virologically suppressed (defined as three consecutive HIV-1 RNA values <50 copies/mL) [24]. 73% of a French cohort on a protease inhibitor-containing regimen for 12 years (n = 229) had “prolonged virological suppression” (VL <500 copies/mL three times in 8 months) that was associated with high ART adherence in the first 4-months of treatment [25]. Additionally, a recent report on patients from other regions in Myanmar using routinely collected data presented a cumulative hazard of virological failure at 5 years after starting ART of 17%, and of 22% after 10 years, as well as a low rate of switching to a second-line regimen but using various definitions of virological failure through the time registered for the study [26].

Our genotyping results correspond to previous reports for the country and the region [27] [28], showing that the primary subtypes circulating in the area are recombinant forms. In our patients, all samples were classified as CRF 01_AE. Resistance mutations found were also expected for each genoma region analysed, particularly when considering the drugs used to target HIV-1 [29]. The small number of patients with resistance mutations, however, made further analysis of viral variations not possible.

### Co-morbidities

In our study, we found low prevalence of the co-morbidities studied except for a high rate of past or present tuberculosis infection (48.5% of the sample) which is highly prevalent in Myanmar and also associated with higher attrition rates for people on ART [12]. We also found biochemical alterations represented by high rates of hypertriglyceridemia and ALAT values above the limit of detection.

Both HBV and HCV infections are associated with more rapid progression of liver fibrosis in the event of HIV co-infection. Liver pathology has now been identified as a leading cause of death among HIV patients in high-income countries [30]. Yet, in most LMIC, management and monitoring of hepatitis B and C infection is not integrated into public ART programs and hepatic infections often remain undiagnosed [31]. In our cohort, 13.7% of patients had HCV antibodies and 7.5% were positive for HBsAg which was similar to other HIV/Hepatitis coinfection data for Myanmar showing seroprevalence rates of 5.3% (HCV) and 8.7% (HBV), respectively [32]. Specific risk factors for HCV coinfection were not explored in our study, but an association between fishing as an occupation, unsafe injection practices, and high-risk sexual behaviour have been previously reported in the region [33].

AIDS and ART interact with traditional risk factors associated with some chronic diseases, and complex interactions involving inflammatory effects of HIV and potential anti-retroviral toxicity have been documented in high-income countries. Our study shows rates of cardiovascular morbidity, such as CAD and myocardial infarction, much lower than in other studies in both HIC and LMIC settings, including major research such as the SMART and ESPRIT studies [34] [7]. Comparisons should be made with caution, however, because there can be a survival bias as we assessed only those who were still alive at the time of survey.

Additionally, 17.9% of our patients were diagnosed with hypertension. Recent reports from Myanmar showed that prevalence in general rural population was 39.7% (defined as >140 mmHg for systolic and >90 for diastolic, taking an average of two measurements) [35]; previous analysis from HIC linked a higher prevalence of systolic hypertension with prolonged ART use [36]. The prevalence of AIDS-related malignancies was lower in our cohort when compared to other studies [7]. The prevalence of renal insufficiency in this cohort was similar when compared to other studies in LMIC [37]. However, the prevalence of cirrhosis was higher in our cohort than in other studies, probably related to higher hepatitis B and C co-infection [5].

In HICs, some behavioural chronic disease risk factors, such as tobacco smoking, occur more frequently in HIV-positive populations [38] [39]. However, the proportion of daily smoker in our cohort is similar to the general rural population reported for the country (27%) [35]; of note, despite the high rates of smoking in our cohort, we did not find much cardiovascular disease in the participants studied. Recently, an Asian review [40] showed that in simulated interventions, smoking cessation had the greatest impact in reducing cardiovascular risk and closely approximated the impact of switching from abacavir to an alternate antiretroviral in the reduction of 5-year myocardial infarction risk. In our population, tobacco smoking was common (30% of the subjects) and over 95% of the smokers in the study were male. Alcohol consumption was similarly reported almost exclusively in males (97.9%) reflecting the regional social context and highlighting opportunities for further intervention.

Finally, we observed that 5.7% of the patients developed hypercholesterolemia and 37.1% hypertriglyceridemia, much different than the 13.7% and 6.2% rates found, respectively, in a Thai ART toxicity study over a median 3.7 years [41], and also different than the 47.3% and 19.1% found in the general rural population in Myanmar [35]; probably related with both longer exposure to ART as well as different exposure to risk factors not considered in this study.

Our study is not without limitations. First, the study population is smaller than those found in similar research. Additionally, incomplete CD4 data at baseline prevented us from including it in mortality analysis. VL was only checked at one time point, multiple measurements were not followed in case of detectable VL. Furthermore, other co-morbidities like depression or osteoporosis were not evaluated, nor were biomarkers that have been recently associated with mortality [42], or conditions associated with CAD such as carotid wall thickness, etc. Virological and immunological outcomes and other comorbidities can be overestimated or underestimated as we only included those who survived for more than 9 years. Bias related to the self-reporting of some data (particularly socially stigmatized information) is possible.

## Conclusions

In this cohort, retention in care after 9 to 11 years on ART was high at 68%. Attrition was mainly due to high mortality, particularly earlier mortality at 3 and 12 months after ART initiation, associated with presenting to care with late stage AIDS. Moreover, this study provides further evidence that good long term immunological and virological outcomes in low resources settings are possible, even for patients starting ART with low CD4 values and with a high proportion of patients with current or previous co-infections like tuberculosis. Despite an aging cohort of patients with long-time ART exposure, appropriate clinical outcomes were largely seen.

As individuals with HIV are now living longer, chronic disease and NADEs become of paramount importance, yet studies from LMIC on these comorbidities remain limited and further research is needed [43]. The implications of an ageing HIV infected population with chronic conditions should be addressed in a model that ensures continued access to care in LMIC [44]. Late presentation related to low CD4 cell counts requires further attention. Beyond quickly expanding the quantity of facilities offering ART, other options such as community based treatment support to provide quality treatment, earlier HIV testing, and chronic disease management should also be explored.

## Supporting information

S1 File LTO_DWI_PlosF. Database of collected participant’s information.
(XLSX)
